# Safety and Efficacy Evaluation of Traditional Chinese Medicine (Qingre-Lishi-Yishen Formula) Based on Treatment of Regular Glucocorticoid Combined with Cyclophosphamide Pulse in Children Suffered from Moderately Severe Henoch–Schonlein Purpura Nephritis with Nephrotic Proteinuria

**DOI:** 10.1155/2020/3920735

**Published:** 2020-01-27

**Authors:** Lirong Fan, Huimin Yan, Xiaofang Zhen, Xiaoming Wu, Jing Hao, Linyi Hou, Lei Han

**Affiliations:** Department of Traditional Chinese Medicine, Beijing Children's Hospital, Capital Medical University, National Centre for Children's Health, Beijing 100045, China

## Abstract

**Objective:**

At present, the most appropriate management of Henoch–Schonlein purpura nephritis (HSPN) with nephrotic-range proteinuria still remains controversial; thus, the purpose of this study is to evaluate safety and efficacy of traditional Chinese medicine (TCM), Qingre-Lishi-Yishen Formula (QLYF), integrated with regular oral glucocorticoid and cyclophosphamide intravenous pulse therapeutic regimen in children suffered from moderately severe HSPN with nephrotic proteinuria.

**Methods:**

From 1 January 2012, to 1 January 2016, totally 150 hospitalized children suffered from HSPN with nephrotic proteinuria were included. All were treated with glucocorticoid and cyclophosphamide, and 100 of them were treated with integrative traditional Chinese decoction QLYF. Patients were followed up for 2 years. Rate of adverse event occurrence, short-term clinical effects, long-term clinical effects, and TCM therapeutic evaluation were all compared.

**Results:**

Total adverse event rate was lower in the QLYF group (*χ*^2^ = 5.357, *p* = 0.022); rates of respiratory infection, urinary infection, poor appetite, hepatotoxity, cardiotoxicity, and neutropenia were all decreased in patients who received QLYF (*p* = 0.022); rates of respiratory infection, urinary infection, poor appetite, hepatotoxity, cardiotoxicity, and neutropenia were all decreased in patients who received QLYF (*p* = 0.022); rates of respiratory infection, urinary infection, poor appetite, hepatotoxity, cardiotoxicity, and neutropenia were all decreased in patients who received QLYF (*p* = 0.022); rates of respiratory infection, urinary infection, poor appetite, hepatotoxity, cardiotoxicity, and neutropenia were all decreased in patients who received QLYF (*p* = 0.022); rates of respiratory infection, urinary infection, poor appetite, hepatotoxity, cardiotoxicity, and neutropenia were all decreased in patients who received QLYF (*p* = 0.022); rates of respiratory infection, urinary infection, poor appetite, hepatotoxity, cardiotoxicity, and neutropenia were all decreased in patients who received QLYF (

**Conclusion:**

Compared with merely using regular oral glucocorticoid plus cyclophosphamide pulse therapeutic regimen, the therapeutic regimen that integrates QLYF with the abovementioned western medicine might be a safe means to decrease the occurrence rate of adverse events and improve short-term and long-term clinical effects in children who suffered from moderately severe HSPN with nephrotic proteinuria.

## 1. Introduction

Henoch–Schonlein purpura (HSP) is an immunoglobulin A- (IgA-) mediated disease characterized by a generalized vasculitis mainly involving the skin, joints, gastrointestinal tract, and kidneys [1, 2]. Skin purpura and other extrarenal symptoms usually resolve rapidly without severe complications. However, the long-term prognosis of HSP mainly depends on the severity of renal involvement, termed Henoch–Schönlein purpura nephritis (HSPN) [3, 4]. In one 20-year follow-up study, HSPN leads to the chronic kidney disease (CKD) in up to 20% affected children; furthermore, the ratio could be as high as 40% for children initially expressed as the moderately severe HSPN with nephrotic-range proteinuria [5]. Therefore, effective therapeutic interventions are considered necessary to prevent progressing to end-stage renal disease (ESRD).

According to the *Kidney Disease:* Improving Global Outcomes (KDIGO) guidelines, in HSPN patients with persisting proteinuria of >1 g/day/1.73 m^2^ and glomerular filtration rate (GFR) > 50 ml/min, a 6-month course of glucocorticoid therapy is recommended; in HSPN patients with nephrotic-range proteinuria, standard therapeutic regimen including regular oral glucocorticoid treatment and intravenous cyclophosphamide pulse is recommended [6]. However, it has been demonstrated that glucocorticoid usage could not prevent renal involvement in HSP [7]. Furthermore, the immunosuppressant usage may exert therapeutic effect but may also be blamed for severe adverse events both in the short-term and in the long-term. As to glucocorticoid, use of high-doses for a long time may lead to Cushing's syndrome (moon face, weight gain, and centripetal redistribution of fat), secondary diabetes mellitus, hypertension, dyslipidemia, and ocular complication and prone to infections [8]. Cyclophosphamide is one of the most commonly used alkylating agents, which can exert immunosuppressive function by causing cytotoxic and antiproliferative effects on various immune cells. The long-term use of cyclophosphamide may give rise to gastrointestinal effects (nausea and vomiting), liver toxicity, myocardial damage, bone marrow toxicity, bladder toxicity, and gonadal toxicity [9].

TCM has been used to treat pediatric nephropathy for several decades and has been proved to be effective in our previous clinical research [10]. QLYF is based on the clinical experience of professor Pei Xueyi (Traditional Chinese Medicine Department, Beijing Children's Hospital affiliated to Capital Medical University). In Professor Pei's academic view, the “spleen” is always too insufficient to govern movement and transformation in children, resulting in retention of heat and damp interior [11].  Table 1 displays the basic composition of QLYF, whose monarch herbs are *Herba Pteridis Multifidae*, *Herba Achyranthis Asperae*, *Radix Sophorae Flavescentis*, and *Folium Pyrrosiae*. There is a research telling us that oxymatrine, which is one of the effective constituents of *Radix Sophorae Flavescentis*, exerts cardioprotective effect by preventing ventricular remodelling, myocardial hypertrophy, and myocardial fibrosis [12]. Other studies have indicated that *Herba Achyranthis Asperae* not only exhibits promising anti-inflammatory activity but also shows significant gastroprotective activity [13, 14]. Therefore, we performed this clinical research to clarify the safety and efficacy of QLYF combined with western medicine glucocorticoid and cyclophosphamide for the treatment of HSPN children with nephrotic-range proteinuria.

## 2. Materials and Methods

### 2.1. Patients

From 1 January 2012, to 1 January 2016, 150 moderately severe HSPN children with nephrotic proteinuria were enrolled continuously. The diagnosis of HSPN with nephrotic proteinuria is based on the evidence-based guideline for diagnosis and treatment of HSPN revised by the Kidney Group of Chinese Pediatric Society, Chinese Medical Association [15]. The inclusion criteria were as follows: (1) age of 5–16 years; (2) 24 hour proteinuria ≥50 mg/kg; (3) urine erythrocyte ≥10/HPF; (4) the first-visit TCM pattern is “dampness-heat accumulation syndrome” [16]; (5) therapeutic regimen is glucocorticoid plus cyclophosphamide; (6) patients have no surgical operation history caused by HSP. The exclusion criteria were as follows: (1) during therapeutic period change for or add with another immunosuppressant (cyclosporin A, tacrolimus, mycophenolate mofetil, etc.); (2) diagnosed with hypercalciuria; (3) diagnosed with other systemic disease (systemic lupus erythematosus, ANCA vasculitis etc.) that may influence the renal function; (4) irreversible decrease of renal function within 3 months of disease onset; (5) those with other organs disfunction (liver, heart, brain, etc). Finally, we enrolled the total 182 patients who received regular oral glucocorticoid and cyclophosphamide pulse therapy, of whom 119 also received QLYF (QLYF group) and 63 received merely the western medicine (control group).

### 2.2. Treatment Protocol

#### 2.2.1. Western Medicine Treatment Protocol

All participants received regular oral glucocorticoid plus intravenous cyclophosphamide pulse regimen. Prednisone is commenced at a dose of 1.5–2 mg/kg/d daily (the maximum dose should not exceed 80 mg/d) for 4–8 weeks and followed by 1.5–2 mg/kg/d on alternative days for further 4–8 weeks. On the basis of regularly oral prednisone, cyclophosphamide is intravenous infused at 500–750 m^2^/kg once a month for 6 months and the total cumulated amount of cyclophosphamide ≤150 mg/kg. Finally, oral prednisone dose was gradually reduced to zero by regular outpatient visits. Participants of the QLYF group received the same glucocorticoid and cyclophosphamide regimen as the control group did.

#### 2.2.2. Traditional Chinese Medicine Treatment Protocol

Traditional Chinese herbal decoction QLYF includes basic herbs and limited optional herbs. Basic herbs are specifically targeted for dampness-heat accumulation syndrome, while optional herbs are targeted for variable secondary TCM syndromes in recovery phase of HSPN. Although there were herb adjustments, the core pathogenesis was consistent and the adjusted herbs did not change the overall nature of the QLYF. Basic herbal composition, which could clear damp-heat, nourish kidney, and consolidate essence, is displayed in Table 1. For primary dampness-heat accumulation syndrome, we used TCM syndrome score scale [10] to evaluate the therapeutic effect at baseline, 12-month, and 24-month treatment. The more TCM syndrome score was obtained, the worse the TCM therapeutic effect the participants had. Bian-Zheng-Lun-Zhi (syndrome differentiation and treatment) would be performed according to the “Diagnostics of Traditional Chinese Medicine” and “Pediatrics of Traditional Chinese Medicine” by an experienced TCM doctor at the fixed visit time point. On the basis of the basic herbs, the doctor would adjust herbs according to the “Guidelines for the Diagnosis and Treatment of Pediatric Common Diseases in Traditional Chinese Medicine” recommended when one patient was mixed with other secondary TCM syndromes in the recovery phase. Rules are as follows: (1) for yang deficiency syndrome, *Radix Astragalus* (Huang Qi), *Atractylodes macrocephala Koidz* (Bai Zhu), and *Cortex Cinnamomi* (Rou Gui) would be added; (2) for yin deficiency with effulgent fire syndrome, *Cortex Phellodendri* (Huang Bai) and *Rhizoma Anemarrhenae* (Zhi Mu) would be added; (3) for heat-toxin congestion syndrome, *Rhizoma Smilacis Glabrae* (Tu Fu Ling) and *Herba Hedyotidis Diffusae* (Bai Hua She She Cao) would be added; (4) for blood stasis syndrome, *Semen Persicae* (Tao Ren), *Angelica sinensis* (Dang Gui), and *Thizoma chuanxiong* (Chuan Qiong) would be added; (5) for phlegm-damp syndrome, *Pheretima* (Di Long), *Bombyx Batryticatus* (Jiang Can), and *Periostracum Cicadae* (Chan Yi) would be added; (6) for spleen-deficiency and qi-stagnation syndrome, *Poria cocos* (Fu Ling), *Radix Aucklandiae* (Mu Xiang), and *Fructus Amomi* (Sha Ren) would be added. Raw herbs were all provided by Cachet Pharmaceutical Co., Ltd (Beijing, China). The herbs were initially soaked in water for 1 hour at room temperature, followed by 1 hour of decoction at 100°C, then filtering the solid components of the residue, and finally condensing the decoction to the specified volume. The herbal formula preparation was completed by the decoction room of traditional Chinese medicine in the Pharmacy Department of Beijing children's hospital. The formula was taken orally twice a day, a combination of gastric volume and drug tolerance was considered, and drug volume is set as the 50 ml for patients weighing <20 kg and 100 ml for the patients weighing ≥20 kg. TCM treatment lasted for about 2 years.

### 2.3. Data Collection

Demographic data and clinical variables during the first hospitalization were collected. There were gender, age, clinical manifestation, systolic blood pressure, serum albumin, serum urea, serum creatinine, eGFR, triglyceride, cholesterol, urinary erythrocyte count, 24 hour proteinuria quantity, serum IgA, serum complement C3, and serum complement C4. After the last cyclophosphamide pulse therapy, patients were assessed by regular outpatient visit once a month for initial half year and every three months until the end of 24 months and overall follow-up lasted for 2 years. Observed indexes included urinary sediment, 24 hour proteinuria, blood routine test, blood biochemistry, TCM syndrome score, and adverse event occurrence during follow-up. Furthermore, the transformation of each patient's secondary TCM syndrome type was analysed by the experienced TCM doctor 6-month, 12-month, 18-month, and 24-month visits.

### 2.4. Study Design

**Figure 1:**
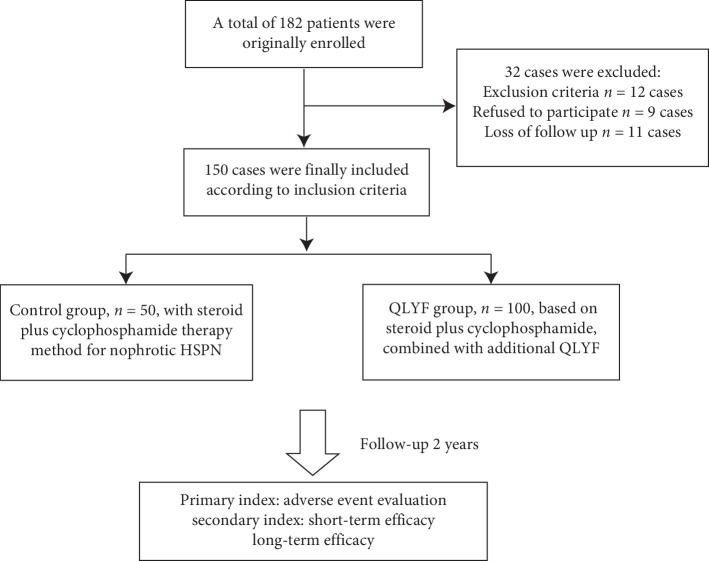
Schematic diagram of the clinical research.

This is a prospective controlled open-label study performed in a single center.  Figure 1 shows schematic diagram of the clinical research. The selection of therapeutic regimen was decided by patients and/or their parents. They considered their compliance and preference, because TCM tastes bitter and should be often adjusted by syndrome differentiation in different periods of HSPN. At the end of follow-up, there left 100 patients in the QLYF group and 50 patients in the control group. We set up a specialized team which was composed of experienced doctors to evaluate the primary and secondary observed indexes.

### 2.5. Primary and Secondary Observed Indexes

The primary observation index is the rate of adverse events, which includes rate of respiratory inflammation, urinary infection, poor appetite, hepatotoxity, cardiotoxicity, infectious diarrhea, neutropenia, and others (including the rare adverse events such as fungal infection, secondary hypertension, secondary diabetes mellitus, secondary ocular hypertension, and dyslipidemia).

The secondary observation index is the short-term and the long-term clinical effect. Short-term clinical effect is reflected by serum creatine, urine, 24 hour proteinuria, and urine red blood counts (URBCs); the abovementioned data were recorded after 3 and 6 months of treatment. According to the book named “Chinese Traditional Medicine New Drug Clinical Research Guiding Principle,” long-term clinical effect is reflected as follows: (1) clinical control: 24 hour proteinuria returned to be normal (≤150 mg) for proteinuria and URBC returned to be normal (≤3/HPF) for hematuria; (2) clinical efficacy: 24 hour proteinuria reduction ≥50% for proteinuria and URBC reduction ≥50% for hematuria; (3) recurrence: by the routine urine test, urine protein turned positive from negative or URBC turned ≥10/HPF from ≤3/HPF. The primary TCM syndrome score is evaluated after 12-month and 24-month treatment, and secondary TCM syndrome distribution was evaluated every 6 month.

### 2.6. Statistical Analysis

The SPSS version 22.0 software (SPSSInc., Chicago, IL, USA) was used to analyse data. Baseline comparisons between the two groups were performed using Student's *t* test for continuous variables, chi-square test for categorical variables, and Mann–Whitney *U* test for nonnormal distribution. Quantitative data were expressed as the mean ± standard deviation (mean ± SD). Differences in measurement data were compared using the chi-square test. Discontinuous variables were expressed as median (Q1, Q3). Values of *p* < 0.05 were considered statistically significant.

## 3. Results

### 3.1. Basic Clinical Characteristics for all Patients

As shown in Table 2, the present study included a total of 150 patients, with 50 cases in the control group (mean age 9.32 ± 2.68, male proportion 52%) and 100 cases in the QLYF group (mean age 9.52 ± 2.96, male proportion 56%). No statistically significant differences in gender, age, additional symptom, systolic blood pressure (SBP), albumin, serum urea, serum creatine, estimated glomerular filtration rate (eGFR), triglyceride, cholesterol, urine red blood cell count (URBC), 24 hour proteinuria, serum IgA, serum C3, and serum C4 were observed between the two groups.

### 3.2. Comparison of Adverse Events of Two Groups

As shown in Table 3, compared with the control group, the total adverse events rate is significantly lower in the QLYF group (66% versus 84%, *χ*^2^ = 5.357, *p*=0.022). Compared with the control group, significantly fewer patients in the QLYF group suffered from respiratory infection (37% versus 58%, *χ*^2^ = 5.966, *p*=0.023), urinary infection (11% versus 26%, *χ*^2^ = 5.580, *p*=0.031), poor appetite (10% versus 26%, *χ*^2^ = 6.573, *p*=0.015), hepatotoxicity (18% versus 34%, *χ*^2^ = 4.770, *p*=0.040), cardiotoxicity (18% versus 36%, *χ*^2^ = 5.921, *p*=0.035), neutropenia (0% versus 6%, *χ*^2^ = 3.444, *p*=0.036), and others(refers to rare adverse events, 7% versus 20%, *χ*^2^ = 5.606, *p*=0.027).

### 3.3. Comparison of Short-Term Renal Indexes between Two Groups

To evaluate the short-term efficacy of QLYF-integrated treatment on HSPN with nephrotic proteinuria, we used renal indexes such as urea, serum creatine, URBC, and 24 hour proteinuria to compare the short-term efficacy of two groups of patients. As shown in Table 4, firstly, we made a comparison within the group. Results indicated that, in both two groups, values of 24 hour proteinuria and URBC significantly decreased after the treatment as compared with those before treatment (Table 2, *p* < 0.05). Meanwhile, compared with baseline data, no significant differences were found in serum creatine and BUN after 3-month or 6-month treatment. And, we made a comparison between two groups in the same treatment time point. Compared with two groups after 3-month treatment and 6-month treatment, only 24 hour proteinuria and URBC were significantly lower in the QLYF group than those in the control group (*p* < 0.05). These results indicated that, as for short-term clinical efficacy, QLYF integrated therapy could decrease 24 hour proteinuria and URBC better in children with nephrotic proteinuria.

### 3.4. Comparison of Therapeutic Evaluation for Two Groups at the End of 2-Year Follow-Up

As shown in Table 5, clinical control rate and effective rate of both hematuria and proteinuria in the QLYF group were better than those in the control group. While both of the clinical control rate (89% versus 74%, *χ*2 = 5.580, *p*=0.031) and effective rate (94% versus 80%, *χ*2 = 6.856, *p*=0.012) on haematuria in the QLYF group have significant difference than those in the control group, there were no significant differences on both of them in proteinuria between the two groups. The recurrence rates both on haematuria (15% versus 36%, *χ*2 = 8.566, *p*=0.006) and proteinuria (7% versus 20%, *χ*2 = 5.606, *p*=0.027) of the QLYF group were significantly better than that of the control group.

### 3.5. Traditional Chinese Medicine Syndrome Therapeutic Effect

As indicated in Table 6, in both two groups, compared with the baseline data, for dampness-heat accumulation syndrome, the TCM syndrome score was lower after 12-month treatment (*p* < 0.05) as well as after 24-month treatment (*p* < 0.05). Compared with the control group at the same follow-up time point, the primary TCM syndrome score was significantly decreased in QLYF group after 12-month treatment (*p*=0.005) as well as after 24-month treatment (*p*=0.001).

**Figure 2:**
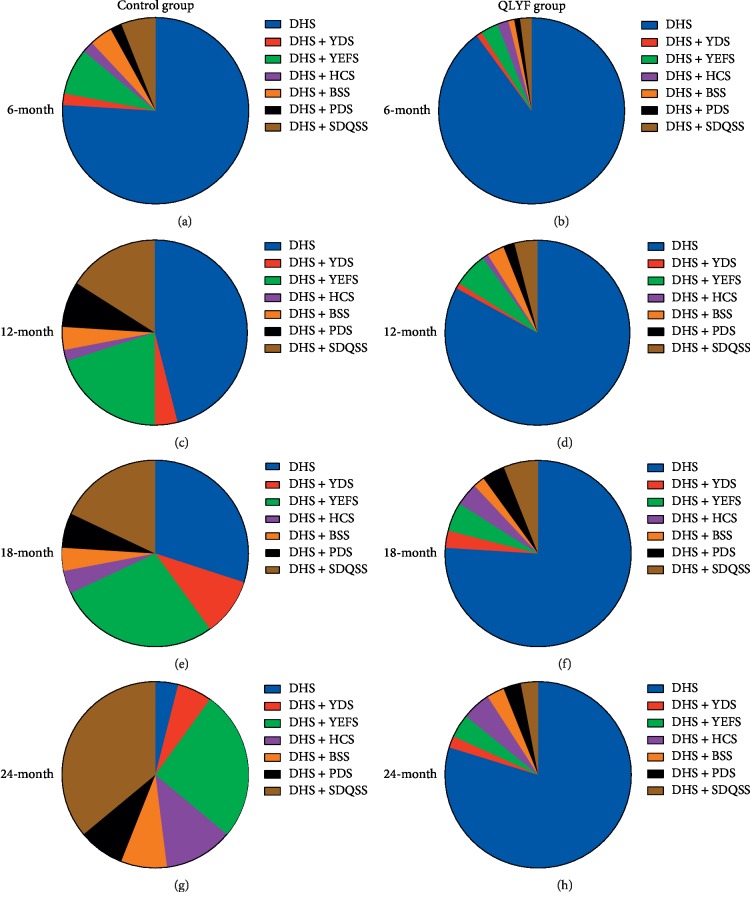
Participants' traditional Chinese medicine syndromes distribution in two groups at the different time points. DHS: dampness-heat accumulation syndrome. YDS: Yang deficiency syndrome. YEFS: Yin deficiency with effulgent fire syndrome. HCS: heat-toxin congestion syndrome. BSS: blood stasis syndrome. PDS: phlegm-damp syndrome. SDQSS: spleen-deficiency and qi-stagnation syndrome.

As Figure 2 shows, in the control group, we could find that the proportion of dampness-heat accumulation syndrome mixed with yin deficiency with effulgent fire syndrome was obviously increased with the extension of follow-up time. We could also find in the control group that the proportion of dampness-heat accumulation syndrome mixed with spleen-deficiency and qi-stagnation syndrome was also increased as the time went on. At the 6-month treatment, dampness-heat accumulation syndrome mixed with yin deficiency with effulgent fire syndrome account for the main part of all, whereas at the 24-month treatment, dampness-heat accumulation syndrome mixed with spleen-deficiency and qi-stagnation syndrome account for the largest of all. However, in the QLYF group, the pie chart shows that the largest proportion always falls on dampness-heat accumulation syndrome, and there are no obvious differences in proportion of dampness-heat accumulation syndrome mixed with any other TCM syndromes as time went on.

## 4. Discussion

As a most common vasculitis in children, HSP is generally a self-limiting condition, usually resolving within 6–8 weeks [17]. However, once HSPN is diagnosed, the HSP prognosis is always dependent on the severity and long-term outcome of HSPN [18]. The HSPN clinical manifestations could be classified as follows: (1) isolated hematuria; (2) isolated proteinuria; (3) hematuria and proteinuria; (4) nephritic syndrome; (5) nephrotic syndrome; (6) rapidly progressive glomerulonephritis; (7) chronic nephritis [19]. One study has indicated that long duration time of nephrotic-state is an independent risk factor of long-term poor prognosis outcomes in children suffered from moderately severe HSPN with nephrotic proteinuria [20]. Hence, it is necessary to perform early aggressive treatment when the HSPN is at early progress stage. A recent meta-analysis has showed that HSPN patients who received immunosuppressive agents plus regular glucocorticoid treatment have better complete remission rate and total remission rate than those who received merely regular glucocorticoid treatment. Furthermore, children seem to benefit more from immunosuppressive agents plus glucocorticoid treatment than adults [21]. HSP recurs in about 1/3 of affected children, and the rate appears to be more common in older children and in children with renal involvement [22]. However, there seems that glucocorticoid might have no direct relevance with preventing the renal involvement [23], and there exists some inevitable adverse events using glucocorticoid and other immunosuppressive agents.

To evaluate the efficacy and advantage for integrated QLYF with the western medicine treatment, we performed this prospective controlled study. It could be inferred from the results that QLYF integrated with western medicine treatment has advantages over merely western medicine treatment in improving short-term efficacy, reducing HSPN recurrence (both proteinuria and hematuria) and preventing adverse events (both common and rare). Furthermore, in academic perspective of TCM, on the one hand, QLFY could decrease more dampness-heat accumulation syndrome score than the control group. On the other hand, long-term use of immunosuppressive agents may cause many secondary TCM syndromes, especially yin deficiency with effulgent fire syndrome and spleen-deficiency and qi-stagnation syndrome, while QLYF-integrated treatment could effectively prevent the occurrence of secondary TCM syndrome in recovery phase of HSPN.

A recent study found that the presence of hematuria was closely associated with a faster decrease in renal function in advanced massive proteinuric patients, especially in younger patients with high levels of proteinuria [24]. Therefore, it is necessary to alleviate hematuria especially when one patient is in a massive proteinuria state. However, although massive proteinuria could be treated by steroids and immunosuppressant, there is lack of disease-targeted and excellent western medicine choice for coinstantaneous haematuria. Previous clinical studies have demonstrated that TCM could exert better clinical effect on haematuria than merely using western medicine [10, 25].

At present, more attention has been focused on TCM with its well-defined and established therapeutic system. TCM posits that disease of the body arises from an imbalance within the body and between the body and the nature, leading to an alteration in the entire body system [26]. In the practice of TCM, it is generally considered that multiple herbal medications are more effective than a single herbal agent, and in this way, the TCM formula could increase or promote therapeutic effectiveness and minimize toxicity and side effects [27].

In Prof. Pei's academic view, the essence of HSP is intertwined damp-heat and consumptive moving “blood”, the disease position is mainly in the “lung,” “spleen,” and “kidney,” the cause could be ascribed to “wind,” “heat,” “dampness,” “stasis,” and “deficiency.” HSP always occurs in later period of epidemic febrile disease, when dampness-heat toxin still exists and accumulating in blood tier, thus injuring the channels and collaterals, with blood-heat bleeding; above all, HSP belongs to “dampness-syndrome” and “blood-syndrome.” Haematuria appearance is considered to be due to dampness-heat accumulation, thus damaging the liver and kidney. In detail, the mechanism is that dampness-heat has accumulated inside for a long time and then flows into the lower Jiao, damaging the liver and kidney, injuring yin collaterals, finally blood being failed to circulate in the vessels. In our herbal formula, some constitutes such as Lian Qiao, Chi Xiao Dou, and Dan Dou Chi could clear the dampness-heat in lower Jiao, thus cooling the blood for hemostasis. For patients with intractable and protracted haematuria, constitutes such as Pu Huang could warm and smooth channels and collaterals, thus consolidating the lower Jiao aiming at hemostasis [28]. QLYF prescription is based on the above principles.

Different from single-target therapy of western medicine, traditional Chinese herbal formula is usually a combination of prescriptions and multiple targets for disease. QLYF basic components totally contain fifteen kinds of herbs, and they play different roles in the therapy process. In the QLYF basic components, monarch herbs are *Radix Sophorae Flavescentis*, *Folium Pyrrosiae*, *Herba Achyranthis Asperae*, and *Herba Pteridis Multifidae*. *Radix Sophorae Flavescentis*, which is bitter and cold in nature, takes its unique effect by heat-clearing and damp-drying. *Folium Pyrrosiae* keeps water unobstructed up to bladder. *Achyranthis Asperae* and *Herba Pteridis Multifidae* play mutual roles in dispersing the dampness-heat and cooling blood to stop bleeding. Minister drugs are *Semen Coicis*, *Herba Patriniae Scabiosaefoliae*, *Fructus Forsythiae Suspensae*, *Semen Vignae Angularis*, *Stamen Nelumbinis*, and *Semen Sojae Preperatum*. *Semen Coicis* which could invigorate spleen to eliminate dampness and induce diuresis to reduce edema, combined with *Herba Patriniae Scabiosaefoliae*, takes the synergistic effect in clearing away heat and toxicant. *Fructus Forsythiae Suspensae* dissipates blood stasis and qi; *Semen Vignae Angularis* moves body fluid to diuretics; *Stamen Nelumbinis* strengthens the kidney to stop emission; *Semen Sojae Preperatum* promotes the circulation of qi and blood. Ministerial herbs supplement with each other in removing dampness and promoting diuresis, detoxifying and arresting seminal emission, and dispersing renal qi. QLYF has been used in children suffered from HSPN for a long time. It is proved to be effective not only in previous randomized clinical trial [10] but also in a recent published animal experiment study [29].

According to TCM theory, glucocorticoids are masculine material. Initially, long-term or high-dose glucocorticoids use would generate heat and reinforce yang, making exhaustion of yin fluid and finally resulting in syndrome of hyperactivity of fire due to yin deficiency, Lian Qiao, Shan Yao, and Zhi mu could nourish yin to lessen fire; in glucocorticoid administration gradually decreased process, patients would fall into syndrome of deficiency of both yin and yang, Fu Ling, Ze Xie, Chan Yi, Lian Qiao could nourish yin and warm the kidney [30, 31]. The TCM syndrome distribution variation tendency results in our clinical trial is consistent with the abovementioned theory. Numerous clinical trials have also confirmed that TCM could lessen the glucocorticoid side effect. Li and Dong compared clinical symptoms with or without TCM in nephrotic syndrome, and finally they found that the incidences of flushed face, tachycardia, insomnia, acne, and thrombosis were significantly lower in TCM-integrated group [32]. Xie conducted a clinical trial to compare the adverse events occurrence with or without TCM, and they found that Cushing's syndrome, infection, upper gastrointestinal haemorrhage, and femoral head necrosis were significantly less in the TCM integrated group, and finally they concluded that the TCM-integrated group (10%) has significantly fewer side effects than the control group (50.31%) [33]. Xiang organized a clinical randomized controlled trial to explore the TCM effect in treating primary nephrotic syndrome; this research revealed that TCM-integrated treatment could significantly improve the clinical effect and prevent adverse events (hepatic function damage, hyperglycemia, acne, mental excitation, and central obesity) during glucocorticoid long-time use [34].

In addition to glucocorticoid, the cyclophosphamide also leads to a lot of adverse events, and there are numerous basic experiments demonstrating that some constitutes of our herbal formula could alleviate the adverse events. It has been proved by Zhang et al. that the fruit of Forsythia suspense can effectively inhibit liver fibrosis by abrogating hepatic stellate cells activation, reversing the liver epithelial-mesenchymal transition and reducing liver extracellular matrix deposition in the mouse model [35]. Another in vivo study has indicated that the extract of Forsythia suspense has a potential to develop anti-hyperglycemic and anti-hyperlipidemic agent via regulation of oxidative stress, hepatic glucose metabolism, and pancreatic insulin secretion [36]. Bo et al. have shown that paeoniflorin, which is the main component of Radix Paeoniae Alba, could notably reduce blood pressure variability, stabilize blood pressure, and mitigate target organ damage in spontaneous hypertensive mice [37]. Furthermore, it is proved by Chen et al. that the anti-hypertensive active of Radix Paeoniae Alba extract may be related to its effect on regulating serum nitric oxide and endothelin levels [38]. Oxymatrine, one of the principle components of Radix Sophorae Flavescentis, was demonstrated in vitro and in vivo by Zhang et al. that may be a promising cardioprotective agent in part through inhibition of cardiac apoptosis and oxidative stress [39].

Consistently, our clinical observation indicated that QLYF integrated with western medicine could significantly reduce common adverse events like respiratory infection, urinary infection, poor appetite, hepatotoxity, cardiotoxicity, and leukocytopenia, as well as rare adverse events like fungal infection, secondary hypertension, secondary diabetes mellitus, secondary ocular hypertension, and dyslipidemia.

## 5. Conclusions

In conclusion, this clinical study indicated that, compared with merely western medicine regimen, QLYF integrated with regular oral glucocorticoid and intravenous cyclophosphamide pulse treatment might decrease the adverse events, decrease the TCM syndrome score, prevent the occurrence of secondary TCM syndrome, and improve the short-term (both in hematuria and proteinuria) and the long-term (in clinical control rate, effective rate, and recurrence rate) clinical efficacy in children suffered from HSPN with nephrotic proteinuria.

## Figures and Tables

**Table 1:** Basic herbal composition of traditional Chinese herbal decoction QLYF.

TCM materials	Equivalent pharmaceutical name	Amount (g)
Feng Wei Cao	*Herba Pteridis Multifidae*	15
Yi Yi Ren	*Semen Coicis*	30
Ku Shen	*Radix Sophorae Flavescentis*	10
Shi Wei	*Folium Pyrrosiae*	12
Dao Kou Cao	*Herba Achyranthis Asperae*	15
Bai Jiang Cao	*Herba Patriniae Scabiosaefoliae*	10
Lian Qiao	*Fructus Forsythiae Suspensae*	10
Chi Shao	*Radix Paeoniae Rubra*	10
Shan Yao	*Rhizoma Dioscoreae Oppositae*	15
Pu Huang	*Pollen Typhae*	10
Lian Xu	*Stamen Nelumbinis*	10
Dan Dou Chi	*Semen Sojae Preperatum*	12
Chi Xiao dou	*Semen Vignae Angularis*	30
Qian Shi	*Semen Euryales*	20

**Table 2:** Basic clinical characteristics for all patients.

	Control group (*n* = 50)	QLYF group (*n* = 100)	*p*
Gender (male)	26 (52%)	56 (56%)	0.643
Age (year)	9.32 ± 2.68	9.52 ± 2.96	0.692
*Additional symptom*			
GI involvement	23 (46%)	41 (41%)	0.559
Joint involvement	17 (34%)	21 (21%)	0.127
SBP (mmHg)	108.2 ± 12.6	109.5 ± 11.3	0.543
Albumin (g/L)	38.45 ± 5.21	33.11 ± 6.45	0.204
Urea (mmol/L)	4.45 ± 1.41	4.46 ± 1.52	0.970
Creatine (*μ*mol/L)	46.57 ± 7.24	45.13 ± 7.93	0.283
eGFR(ml/min·1.73 m^2^)	97.5 ± 16.0	99.6 ± 10.2	0.314
Triglyceride (mmol/L)	1.62 ± 0.90	1.44 ± 0.94	0.275
Cholesterol (mmol/L)	4.87 ± 1.11	4.63 ± 1.19	0.235
URBC (/HP)	26.88 ± 11.16	25.92 ± 11.06	0.618
24 h proteinuria (g)	2.76 ± 0.88	2.70 ± 0.73	0.659
Serum IgA (g/L)	1.97 ± 0.68	1.84 ± 0.66	0.278
Serum C3 (g/L)	0.993 ± 0.271	0.991 ± 0.251	0.976
Serum C4 (g/L)	0.732 ± 0.615	0.578 ± 0.459	0.170

GI: gastrointestinal; SBP: systolic blood pressure; URBC: urine red blood cell count; eGFR: estimated glomerular filtration rate; 24 h: 24 hour.

**Table 3:** Comparison of adverse events of two groups.

Adverse events	Control group, *n* = 50	QLYF group, *n* = 100	*χ* ^2^	*p*
Total adverse events rate	42 (84%)	66 (66%)	5.357	0.022
Respiratory infection	29 (58%)	37 (37%)	5.966	0.023
Urinary infection	13 (26%)	11 (11%)	5.580	0.031
Infectious diarrhea	3 (6%)	3 (3%)	0.195	0.401
Poor appetite	13 (26%)	10 (10%)	6.573	0.015
Hepatotoxicity	17 (34%)	18 (18%)	4.770	0.040
Cardiotoxicity	18 (36%)	18 (18%)	5.921	0.035
Neutropenia	3 (6%)	0 (0%)	3.444	0.036
Others^*∗*^	10 (20%)	7 (7%)	5.606	0.027

^*∗*^Rare adverse events, such as fungal infection, secondary hypertension, secondary diabetes mellitus, secondary ocular hypertension, and dyslipidemia.

**Table 4:** Comparison of short-term renal indexes between two groups.

Time point	Group	Creatine (*μ*mol/L)	Urea (mmol/L)	24 h proteinuria (g)	URBC (HP)
Prior treatment	Control group	46.57 ± 7.24	4.45 ± 1.41	2.76 ± 0.88	26.88 ± 11.16
	QLYF group	45.13 ± 7.93	4.46 ± 1.52	2.70 ± 0.73	25.92 ± 11.06
3-month treatment	Control group	43.13 ± 5.85	4.12 ± 1.22	1.37 ± 0.45^*∗*^	12.64 ± 5.78^*∗*^
	QLYF group	41.52 ± 6.01	4.09 ± 1.32	1.20 ± 0.42^*∗*˄^	9.80 ± 4.22^*∗*˄^
6-month treatment	Control group	40.41 ± 5.19	3.75 ± 1.24	0.42 ± 0.24^*∗*#^	5.16 ± 7.52^*∗*#^
	QLYF group	39.14 ± 5.61	3.74 ± 1.28	0.31 ± 0.18^*∗*#˄^	2.46 ± 1.35^*∗*#˄^

^*∗*^ *p* < 0.05 compared with the same group prior treatment; #*p* < 0.05 compared with the same group in 3-month treatment; ^˄^*p* < 0.05 compared with the control group in the same treatment period.

**Table 5:** Comparison of therapeutic evaluation for two groups at the end of 2-year follow-up.

Group	Proteinuria			Haematuria		
	Clinical control rate	Effective rate	Recurrence rate	Clinical control rate	Effective rate	Recurrence rate
Control group	41 (82%)	42 (84%)	10 (20%)	37 (74%)	40 (80%)	18 (36%)
QLYF group	85 (85%)	91 (91%)	7 (7%)	89 (89%)	94 (94%)	15 (15%)
χ^2^	0.223	1.625	5.606	5.580	6.856	8.566
*p*	0.643	0.274	0.027	0.031	0.012	0.006

**Table 6:** Primary TCM syndrome score was compared between two groups at the different time points.

Time	QLYF group	Control group	*Z*	*p*
Baseline	38 (28, 50)	38 (30, 49)	−0.579	0.563
12-month	20 (16, 27.5)^*∗*^	25 (18, 30.5)^*∗*^	−2.780	0.005
24-month	8 (6, 12)^*∗*^	12 (8, 16)^*∗*^	−3.437	0.001

^*∗*^Compared with the 0-week baseline data, after 4-week, and 12-week routinely treatment; the TCM syndrome score was statistically significantly (*p* < 0.05).

## Data Availability

The figures and data used to support the findings of this study are included and available within the article.
